# Prophylactic Administration of Vector-Encoded Porcine Granulocyte-Colony Stimulating Factor Reduces *Salmonella* Shedding, Tonsil Colonization, and Microbiota Alterations of the Gastrointestinal Tract in *Salmonella*-Challenged Swine

**DOI:** 10.3389/fvets.2016.00066

**Published:** 2016-08-25

**Authors:** Shawn M. D. Bearson, Bradley L. Bearson, Crystal L. Loving, Heather K. Allen, InSoo Lee, Darin Madson, Marcus E. Kehrli

**Affiliations:** ^1^Food Safety and Enteric Pathogens Research Unit, National Animal Disease Center, ARS, USDA, Ames, IA, USA; ^2^Agroecosystems Management Research Unit, National Laboratory for Agriculture and the Environment, ARS, USDA, Ames, IA, USA; ^3^Department of Biological Sciences and Biotechnology, Hannam University, Daejeon, South Korea; ^4^Veterinary Diagnostic and Production Animal Medicine, College of Veterinary Medicine, Iowa State University, Ames, IA, USA

**Keywords:** *Salmonella*, granulocyte-colony stimulating factor, swine, immune stimulation, alternatives to antibiotics

## Abstract

*Salmonella* colonization of food animals is a concern for animal health and public health as a food safety risk. Various obstacles impede the effort to reduce asymptomatic *Salmonella* carriage in food animals, including the existence of numerous serovars and the ubiquitous nature of *Salmonella*. To develop an intervention strategy that is non-specific yet effective against diverse *Salmonella* serovars, we explored the prophylactic use of a cytokine to decrease *Salmonella* in swine by boosting the host’s innate immune system. Granulocyte-colony stimulating factor (G-CSF) is the major cytokine regulating the production, differentiation, function, and survival of neutrophils. Neutrophils play a critical role in the response to *Salmonella;* therefore, we evaluated the vectored-delivery of porcine G-CSF as a prophylactic to reduce *Salmonella* in pigs. Crossbred pigs, 5 weeks of age, were intramuscularly injected with a replication-defective human adenovirus (Ad5) engineered to express porcine G-CSF (Ad5-G-CSF, *n* = 9). Control pigs received the same Ad5 vector lacking the gene encoding G-CSF (Ad5-empty, *n* = 7). Four days later, all pigs (*n* = 16) were intranasally inoculated with 1 × 10^7^ colony forming unit (CFU) of *Salmonella enterica* serovar Typhimurium UK1. At 2 and 3 days post-challenge with *Salmonella*, Ad5-G-CSF-treated pigs shed significantly less *Salmonella* (~10^3^ CFU/g) in their feces than Ad5-empty-treated pigs (~10^4^–10^5^ CFU/g; *P* < 0.05). A significant 4-log reduction in tonsil colonization was also observed in the Ad5-G-CSF-treated pigs at 7 days post-challenge (*P* < 0.05). In the gastrointestinal tract, the Peyer’s patch region of the ileum exhibited a significant 0.5-log reduction in colonization in the Ad5-G-CSF-treated pigs (*P* < 0.05). The microbiota of all challenged pigs was assessed by sequencing and analyzing the V1–V3 region of the 16S rRNA gene from fecal DNA samples. The microbial community structure of *Salmonella*-challenged pigs was less disturbed post-challenge in the Ad5-G-CSF-treated pigs than the Ad5-empty-treated pigs. This suggests that Ad5-G-CSF administration mitigated changes in the microbial community structure caused by *Salmonella* challenge. Collectively, these data suggest that delivery of a targeted immunostimulant to enhance neutropoiesis may be a strategy to reduce *Salmonella* colonization, potentially during periods of immunological stress.

## Introduction

Asymptomatically colonized food animals are a major reservoir of the human foodborne pathogen *Salmonella* (1–3). Intervention strategies are needed to not only decrease the overall prevalence of *Salmonella* in food animals but also reduce an animal’s susceptibility to *Salmonella* during times of production stress, such as farrowing, weaning, mixing, and transportation. Controlling *Salmonella* is challenging due to the broad host range, ubiquitous distribution, and number of *Salmonella* serovars (>2,500). To overcome the complexity of *Salmonella*, management strategies that target innate immune mechanisms warrant exploration to control the commensal-like state of this human foodborne pathogen in the gastrointestinal tract of animals contributing to our food (animal) supply.

An animal’s innate immune system offers multiple pathways that can be modulated to fight disease-causing agents without activation of the adaptive immune system, which is the primary target of vaccination strategies. Instead, bolstering an innate immune response during stressful events in animal production or periods of immune dysfunction could reduce pathogen recrudescence and infection susceptibility. One possible intervention to address this vulnerability is the prophylactic use of biotherapeutic proteins, such as cytokines [reviewed in Ref. (4)]. Granulocyte-colony stimulating factor (G-CSF) is a cytokine involved in the production, differentiation, and function of granulocytes (especially neutrophils) from bone marrow (5–7). Neutrophils are phagocytic cells of the innate immune system, and their killing mechanism provides a critical first line of defense against bacterial and viral infections (8). Recombinant human G-CSF (Neulasta, Amgen Inc.) is FDA-approved for use in humans to decrease the incidence of infection in neutropenic patients receiving myelosuppressive anti-cancer drugs (9). Recombinant bovine G-CSF (Imrestor, Elanco) has also been approved by the FDA for use in dairy cattle to restore neutrophil function and neutrophil numbers during periparturient immune suppression (10–13). Previous work by our group established that the delivery of a replication-defective human adenovirus 5 encoding porcine G-CSF increased the number of functional neutrophils in circulation (14), thus demonstrating the potential for modulating the swine immune system by targeting the G-CSF pathway.

Rapid neutrophil influx into the intestines is the hallmark of a *Salmonella* infection (15, 16). In our previous work, cytokines involved in neutrophil production and recruitment were upregulated in swine following *Salmonella* challenge (17, 18). Furthermore, van Diemen et al. demonstrated higher numbers of circulating neutrophils with greater polymorphonuclear neutrophil (PMN) function in pigs bred for resistance to *Salmonella enterica* serovar Choleraesuis (19). Thus, we hypothesized that elevating the abundance of circulating neutrophils in pigs prior to *Salmonella* exposure may assist in controlling *Salmonella* colonization and shedding. The results demonstrate the beneficial effects of Ad5-G-CSF-induced neutrophilia on the reduction of *S. enterica* serovar Typhimurium (*S*. Typhimurium) colonization and shedding in swine, as well as decreased *Salmonella*-induced disturbance of the gastrointestinal microbiota, suggesting prophylactic use of porcine Ad5-G-CSF may serve as a biotherapeutic approach to reduce *Salmonella* in pigs.

## Materials and Methods

### Swine Experiment

Sixteen crossbred, conventionally reared piglets from three *Salmonella*-fecal-negative sows were weaned at 12 days of age and shipped to the National Animal Disease Center, Ames, IA, USA. Siblings from each litter were divided and raised in two isolation rooms. Piglets tested fecal-negative for *Salmonella spp*. twice over a 2-week period using bacteriological culture with selective enrichment (20). At 5 weeks of age, piglets received an intramuscular injection of 10^10^ TCID_50_/pig of a replication-defective human adenovirus (Ad5) engineered to express porcine G-CSF (Ad5-G-CSF, *n* = 9) (14). As previously described, Ad5-G-CSF was derived by directionally cloning G-CSF cDNA into the AdEasyTM XL System (Stratagene, La Jolla, CA, USA) and propagated in specialized AD-HEK-293 cells. Control pigs received the same Ad5 vector lacking the gene encoding G-CSF (Ad5-empty, *n* = 7). Four days later, all pigs (*n* = 16) were intranasally inoculated with 1 × 10^7^ colony forming unit (CFU) of a nalidixic acid-resistant derivative of *S. enterica* serovar Typhimurium UK1 (21) that had been passaged in swine and isolated from the ileocecal lymph node of a pig (strain name: SB 377). Fecal samples were collected at 0, 1, 2, 3, and 7 days post-inoculation (d.p.i.) for microbiota analysis as well as quantitative and qualitative *Salmonella* culture analyses (see below). Blood samples were collected from the jugular vein at −4, −2, 0, 1, 2, 3, and 7 d.p.i. for enumeration of circulating blood cells by flow cytometry (see below). At 7 d.p.i., all pigs were euthanized and necropsied to obtain tissue samples from the tonsil and the intestinal tract (ileal Peyer’s patches, ileocecal lymph nodes, and cecum) for quantitative and qualitative *Salmonella* culture analysis (see below). Procedures involving animals followed humane protocols as approved by the USDA, ARS, NADC Animal Care and Use Committee in strict accordance with the recommendations in the Guide for the Care, and Use of Laboratory Animals of the National Institutes of Health.

### Bacteriology

For quantitative bacteriology, 1 g of pig feces was combined with 5 ml PBS, vortexed, and 0.1 ml directly plated to XLT-4 medium (Beckton, Dickinson and Co., Sparks, MD, USA) containing 30 μg/ml of nalidixic acid. For tissue samples, 1 g of each tissue was combined with 2 ml of PBS in a whirlpak bag, pounded with a mallet, and homogenized in a Stomacher (Seward, Westbury, NY, USA) for 1 min. One hundred microliters of the resulting solution was aliquoted onto XLT-4 medium containing nalidixic acid. One hundred microliters of a 10-fold dilution of each fecal and tissue sample was also plated, and additional dilutions were performed when CFU reached >300/plate. Following 48 h of incubation at 37°C, black colonies were enumerated and a single colony from each plate was confirmed to be *Salmonella* by serogroup antiserum agglutination (Beckton, Dickinson and Co., Sparks, MD, USA). The total number of CFU for each quantitative tissue or fecal sample was calculated per gram of sample by obtaining the number of *Salmonella* per plate and multiplying by the dilution factor.

Qualitative bacteriology of *Salmonella* was performed as follows: 1 g (fecal) or 0.1 ml (homogenized tissue) samples were inoculated in 10 ml tetrathionate broth (TET; VWR, Rutherford, NJ, USA) for 48 h of growth at 37°C. Following incubation, 0.1 ml of each culture was transferred to 10 ml Rappaport–Vassiliadis medium (RV; Difco) and incubated at 37°C for 18–20 h. Cultures were streaked on XLT-4 medium containing nalidixic acid. Colonies suspicious for *Salmonella* were confirmed by serogroup antiserum agglutination.

Statistical analysis for *Salmonella* shedding in feces (CFU/g) was Log10-transformed and analyzed using a mixed linear model for repeated measures (Proc Mixed in SAS for Windows, version 9.2; SAS Institute Inc., Cary, NC, USA). Covariance structures within pigs across time were tested and modeled using the REPEATED statement to determine the optimal covariance structure. Linear combinations of the least-squares mean estimates were used in *a priori* contrasts after testing for a significant (*P* < 0.05) treatment group effect. Comparisons were made between each group at each time point, using a 5% level of significance (*P* < 0.05) to assess statistical differences. The endpoint data for bacterial colonization (CFU/g) of tissues collected at necropsy were Log10-transformed and analyzed by analysis of variance using a general linear model for unbalanced data. A 5% level of significance (*P* < 0.05) was used to assess statistical differences.

### Whole Blood Cell Differential

White blood cell counts were performed via flow cytometry as previously described (14). Briefly, a 50-μl aliquot of anti-coagulated (EDTA) whole blood was added to a tube containing monoclonal antibody to porcine granulocytes (6D10, Serotech, USA) with appropriate secondary fluorochrome-labeled antibody. After a 20-min incubation, cells were fixed and red blood cells lysed with the addition of 1 ml FACS lyse (BD Biosciences, USA). Microbeads (Spherotech, USA) were added to the tube immediately prior to data acquisition on a flow cytometer (BD LSR II, Becton Dickinson, USA). A gate was drawn around the beads and events were collected on each parameter (neutrophil gate was based on forward and side scatter properties and antibody labeling) until the bead event number was 500. A ratio of total counts to bead counts was used to determine the number of neutrophils per microliter of blood. Calculation of statistical significance of neutrophils per microliter of blood by treatment group was performed using a one-way ANOVA with a Dunnett’s Multiple Comparison Test. A 5% level of significance (*P* < 0.05) was used to assess statistical differences.

### 16S rRNA Gene Sequencing and Analysis

Amplicon libraries of the 16S rRNA gene were generated and sequenced according to Kozich et al. (22), with our primers and procedures described previously (23). Briefly, PCRs contained the following: 17 μl AccuPrime Pfx SuperMix (Life Technologies, Grand Island, NY, USA), 5.0 μM each of the primers i5 + V3 and i7 + V1, and 25 ng of fecal DNA. The following PCR conditions were used: 2 min at 95°C, 22 cycles of (20 s at 95°C, 15 s at 55°C, 5 min at 72°C), 72°C for 10 min. Libraries were normalized using the SequalPrep Normalization Plate Kit (LifeTechnologies) and quantified using both Bioanalyzer (Agilent Technologies, Santa Clara, CA, USA) and Kapa SYBR Fast qPCR (Kapa Biosystems, Wilmington, MA, USA). Normalized pools were sequenced using version 3 (300 × 2) chemistry on the MiSeq instrument (Illumina, San Diego, CA, USA) according to manufacturer’s instructions.

Contig assembly, sequence alignment, chimera removal, and non-bacterial sequence removal were performed in the program mothur (version 1.33.3) (24). Sequences that only occurred once or twice across all samples were removed as potentially spurious. Sequences were rarified to 3,000 sequences, clustered into operational taxonomic units (OTUs) at 97% similarity, and analyzed for community metrics, including richness (25), evenness, and diversity. Analysis of similarity (ANOSIM) and non-metric multidimensional scaling (NMDS) analyses were conducted in PAST (26). Additionally, the OTUs were assigned to bacterial taxonomy using mothur’s implementation of the SILVA database (27). One sample from a pig in the Ad5-G-CSF group at day 7 yielded insufficient sequences to be analyzed. The 16S rRNA gene sequences associated with this study were deposited in Genbank under Bioproject PRJNA339155.

## Results

### Both *Salmonella* Typhimurium Challenge and Porcine Ad5-G-CSF Administration Increased Circulating Neutrophils in Pigs

**Figure 1:**
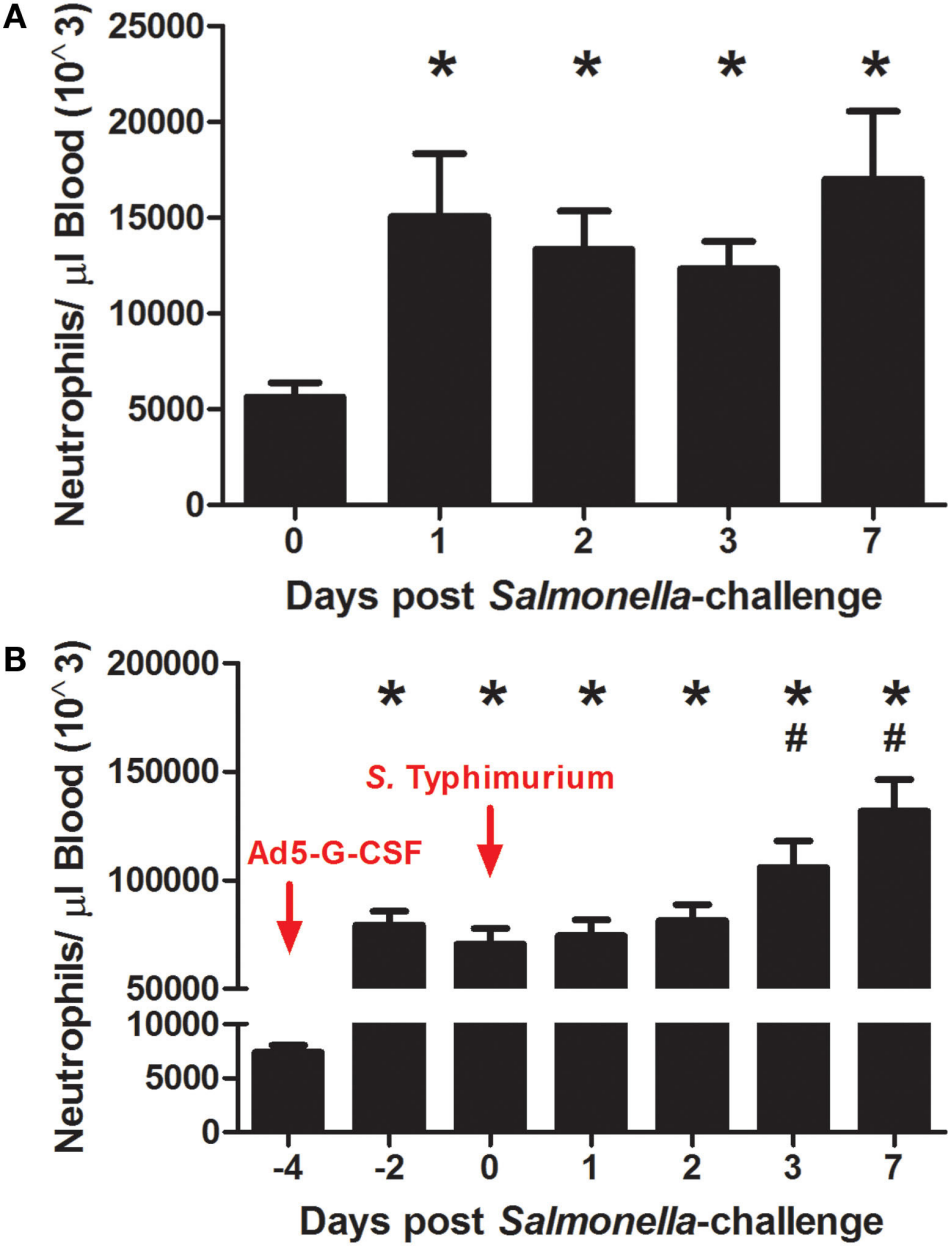
**The number of circulating neutrophils following *S*. Typhimurium challenge and Ad5-G-CSF administration**. Blood samples were collected at the noted day relative to *Salmonella* challenge for neutrophil enumeration by flow cytometry as described in Section “Materials and Methods.” Data are reported as the mean + SEM. **(A)** Pigs were intranasally challenged with 1 × 10^7^ CFU of virulent *S*. Typhimurium UK1 strain (*n* = 7). Significant difference (*P* < 0.05) in circulating neutrophils relative to day 0 (*). **(B)** Pigs received a single intramuscular injection of Ad5-G-CSF followed by S. Typhimurium challenge with 1 × 10^7^ CFU of *S*. Typhimurium UK1 (*n* = 9). Significant difference (*P* < 0.05) in circulating neutrophils relative to day −4 (*) or day 0 (#).

The effects of Ad5-G-CSF administration and *S*. Typhimurium challenge on circulating neutrophils were determined by enumerating neutrophils in the blood at various days after Ad5-G-CSF administration and *S*. Typhimurium challenge. *S*. Typhimurium challenge alone induced a significant approximately threefold increase in circulating neutrophil counts, as values post-challenge were greater when compared to values on the day of challenge (day 0) (Figure 1A). Circulating neutrophils were also enumerated in pigs that were intramuscularly injected with 10^10^ TCID_50_ Ad5-G-CSF prior to *Salmonella* exposure. As expected on the day of *S*. Typhimurium challenge (day 0), which was 4 days after Ad5-G-CSF administration, a significant neutrophilia occurred compared to pre-Ad5-G-CSF numbers (day −4, Figure 1B) or compared to Ad5-empty-treated controls on day 0 (day 0, Figure 1A). Following *Salmonella* challenge of the Ad5-G-CSF group, an additional significant increase in circulating neutrophils was observed at 3 and 7 d.p.i. compared to day 0 (day of *Salmonella* challenge). Collectively, Ad5-G-CSF administration induced a significant and sustained ~10-fold increase in the number of circulating neutrophils, and *Salmonella* challenge also induced significant increases in circulating neutrophils.

### Ad5-G-CSF Treatment Reduced *Salmonella* Fecal Shedding and Tissue Colonization

**Figure 2:**
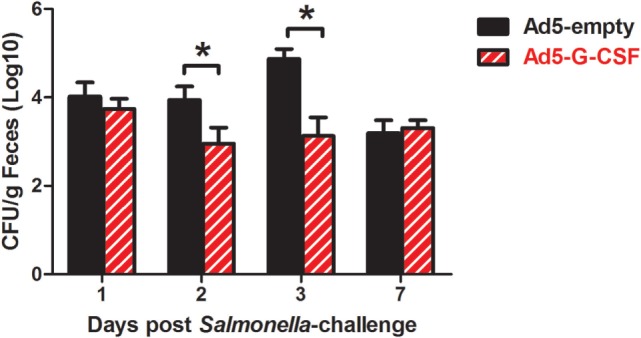
**Fecal shedding from *Salmonella*-challenged pigs, with or without prior Ad5-G-CSF administration**. On day 4 following Ad5-G-CSF or Ad5-empty administration, all 16 pigs were challenged with 1 × 10^7^ CFU of *S*. Typhimurium UK1. *Salmonella* fecal shedding was monitored via bacteriological analysis of fecal samples collected at 1, 2, 3, and 7 d.p.i. *At each timepoint, significant difference (*P* < 0.05) in *Salmonella* CFU/g feces comparing pigs administered Ad5-G-CSF versus Ad5-empty.

**Figure 3:**
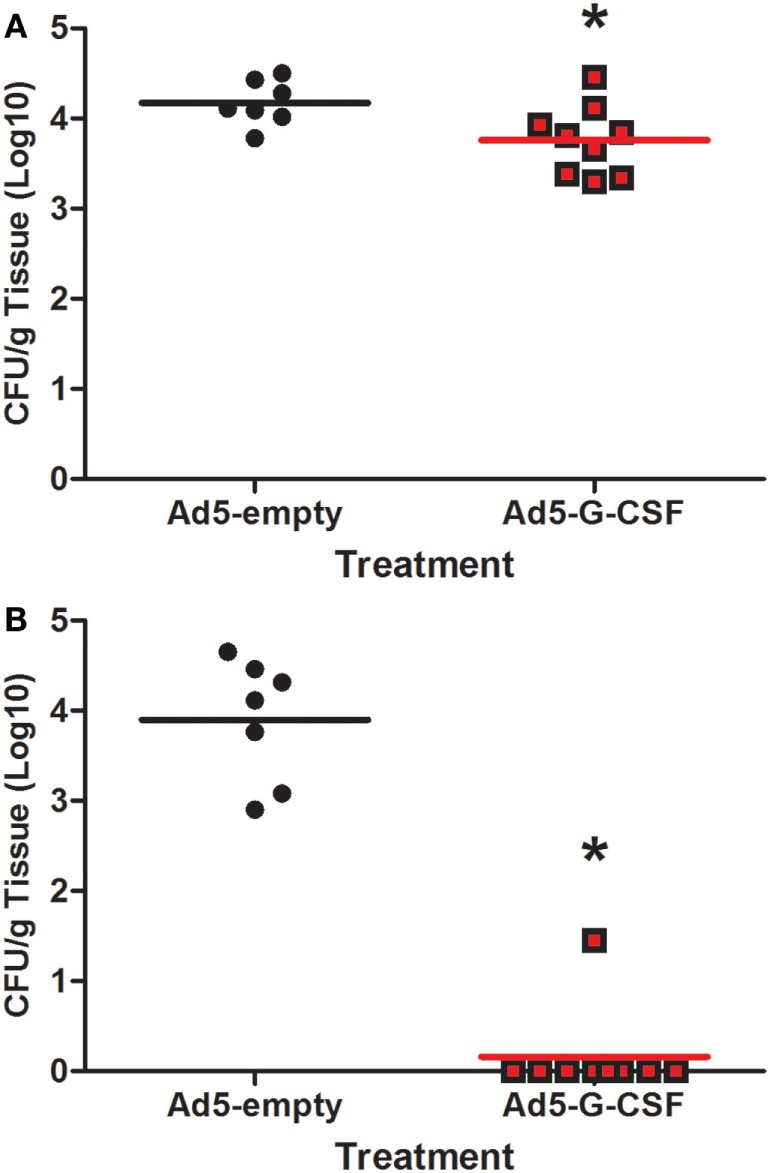
**Tissue colonization from *Salmonella*-challenged pigs, with or without prior Ad5-G-CSF administration**. At 7 d.p.i., *Salmonella* bacteriological analysis (CFU/g) of the **(A)** Peyer’s patch region of the ileum and **(B)** tonsils obtained during necropsy. *Significant difference (*P* < 0.05) comparing Ad5-G-CSF-treated to Ad5-empty-treated pigs at the same time point.

*Salmonella* shedding and tissue colonization were compared between Ad5-G-CSF and Ad5-empty-treated pigs. Ad5-G-CSF-treated pigs shed significantly less *Salmonella* (10^3^ CFU/g feces) when compared to the Ad5-empy-treated pigs at 2 and 3 d.p.i. (10^4–5^ CFU/g feces) (Figure 2). This 1- to 2-log difference between the treatment groups dissipated by 7 d.p.i. as *Salmonella* shedding in the feces of Ad5-empty-treated pigs declined to the level of the Ad5-G-CSF-treated pigs. Typical for swine, a transient fever was observed in the *S*. Typhimurium-challenged pigs, peaking at 2 days post-challenge; no significant difference was observed in the elevated body temperatures between treatment groups (data not shown). Gastrointestinal tissues (ileocecal lymph nodes, Peyer’s patch region of the ileum, and cecum) were analyzed at 7 d.p.i., and all tissues were *Salmonella* positive in both Ad5-G-CSF-treated and Ad5-empty-treated pigs. Of these three tissues, the Peyer’s patch region of the ileum exhibited a significant 0.5-log reduction in *Salmonella* colonization in the Ad5-G-CSF-treated pigs compared to the Ad5-empty-treated group (Figure 3A). A striking difference in tonsil colonization was observed between treatment groups (Figure 3B). Eight of the nine Ad5-G-CSF-treated pigs harbored no detectable *Salmonella* in the tonsil, with only one pig being qualitatively positive for *Salmonella* in the tonsils (i.e., by enrichment). By contrast, all seven Ad5-empty-treated pigs harbored *Salmonella* in the tonsils at an average of ~10,000 CFU/g. These data suggest that prophylactic administration of Ad5-G-CSF can reduce *Salmonella* colonization and subsequent fecal shedding, including the tonsils that have been implicated in the carrier-status of *Salmonella* (28–31).

### The Gastrointestinal Microbiota of *Salmonella*-Challenged Pigs Was More Stable in the Ad5-G-CSF-Treated Pigs

**Figure 4:**
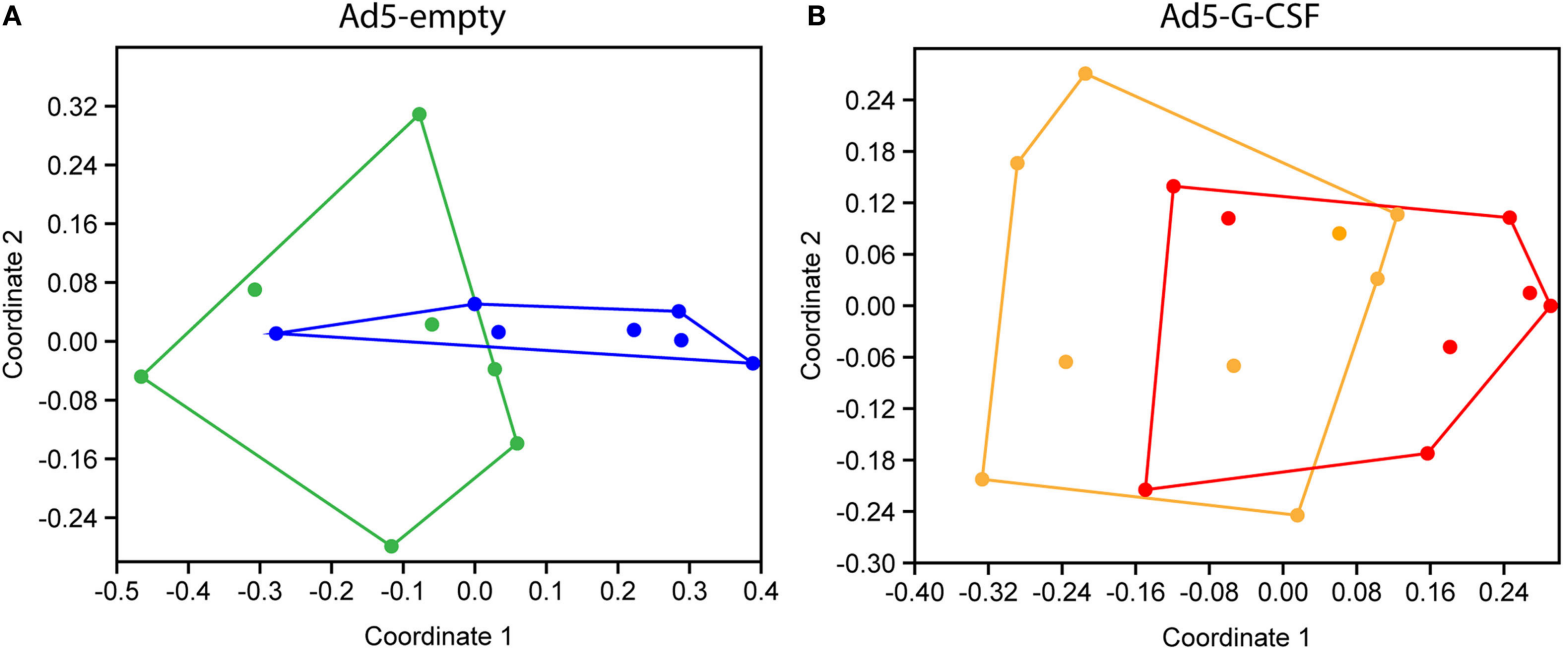
**Non-metric multidimensional scaling (NMDS) analyses of 16S rRNA gene OTUs from *Salmonella*-challenged pigs with prior administration of Ad5-empty (A) or Ad5-G-CSF (B)**. Shown are the fecal microbiotas from samples taken at 3 days [**(A)**, green; **(B)**, orange] and 7 days [**(A)**, blue; **(B)**, red] post-*Salmonella* challenge. OTU cutoff of 97% similarity was used. Stress = **(A)**, 0.1445; **(B)**, 0.1453.

Fecal 16S rRNA gene sequence data were used to compare the gastrointestinal bacterial communities of the Ad5-G-CSF and Ad5-empty treatment groups following *Salmonella* challenge. No significant differences in indices for diversity, evenness, or richness were detected among treatments or timepoints. OTU-based analysis of bacterial community structure showed that at 7 days post-*Salmonella* challenge, the microbiota of pigs that had received Ad5-G-CSF was not significantly different from that of day 2 or 3 (ANOSIM, *p* > 0.05; *R* < 0.1), but the microbiota of pigs that received Ad5-empty treatment was significantly different at day 7 compared to all previous time points (ANOSIM, *p* < 0.05; *R* > 0.25). However, the difference between the Ad5-G-CSF-treated and Ad5-empty-treated groups at day 7 was insignificant. The dissimilarity of the microbiotas between days 3 and 7 was visualized via an NMDS plot, which showed the disturbed microbiota at day 7 in the Ad5-empty-treated animals compared to Ad5-G-CSF-treated animals (Figure 4). These results demonstrate that Ad5-G-CSF administration slightly decreases the beta-diversity changes in the microbiota that are caused by *Salmonella* challenge, suggesting that Ad5-G-CSF mitigated the disturbance to the gut microbiota that was caused by *Salmonella*.

## Discussion

Granulocyte-colony stimulating factor is a cytokine that influences the proliferation, differentiation, maturation, function, and survival of neutrophils (32). Neutrophils are a critical mediator of antimicrobial defense during the initial stages of infection and have effects on a number of microbial targets (8). Circulating neutrophil numbers in swine have been correlated with resistance to salmonellosis, with pigs most resistant to *Salmonella* exhibiting higher numbers of circulating neutrophils and enhanced neutrophil function (19). In the current study, treatment of swine with Ad5-G-CSF increased the number of circulating neutrophils by 10-fold, decreased *Salmonella*-induced disturbance of the gastrointestinal microbiota, and reduced *Salmonella* fecal shedding 1–2 logs during the acute stage of infection. Thus, prophylactic use of G-CSF as an immunostimulant may be an effective strategy to reduce *Salmonella* in swine herds. A farm-to-consumption quantitative microbiological risk assessment (QMRA) for *Salmonella* in pigs in the European Union concluded that interventions should focus on decreasing the level of *Salmonella* in the feces of infected swine because the vast majority of human risk is derived from a subset of pigs with a high concentration of *Salmonella* in their feces (≥10^4^ CFU/g) (33). In our study, the Ad5-empty-treated pigs shed *Salmonella* at 10^4–5^ CFU/g, and Ad5-G-CSF treatment reduced the level of *Salmonella* fecal shedding to 10^3^ CFU/g, further supporting G-CSF administration as a possible risk mitigation strategy.

The dramatic reduction in *Salmonella* colonization of the tonsils in the Ad5-G-CSF-treated pigs also highlights prophylactic treatment with G-CSF as a potential control strategy for persistently infected pigs. *Salmonella* can reside in lymph nodes and especially the tonsils (28–31). In this carrier-state, a stressful event (farrowing, weaning, or transport) can trigger *Salmonella* to re-emerge and reseed the gastrointestinal tract, resulting in shedding recrudescence (34, 35). Reduction of tonsil colonization, as observed in the Ad5-G-CSF-treated pigs, may reduce the reseeding process during stress. An intriguing follow-up study would be to evaluate the recrudescence of *Salmonella* in colonized pigs that are given Ad5-G-CSF prior to an applied stress (mixing, transportation, etc.). While additional investigations of the efficacy and safety of Ad5-G-CSF administration in swine are warranted, our data suggest that increasing the number of circulating neutrophils via Ad5-G-CSF administration may offer a non-specific yet effective method for reducing *Salmonella* colonization in swine.

Inflammation-associated intestinal dysbiosis can result in pathogen expansion, especially for microorganisms, such as *Salmonella*, that are capable of taking advantage of an inflamed environment (36). We have previously shown that *Salmonella* colonization of the porcine gastrointestinal tract causes a disturbance within the gut microbial community (37) and triggers an inflammatory response from the host (17, 18). Intervention strategies that target *Salmonella* during the initial stages of colonization could reduce overall gut inflammation and subsequently prevent the development of a “nutrient-niche” that can be selectively used by *Salmonella* (38). In the current study, prophylactic Ad5-G-CSF administration was beneficial in reducing the *Salmonella*-induced microbiota disturbance. Nevertheless, neutrophils are a primary player in the inflammatory response, and their contribution to the inflammatory response that provides an optimal environment for *Salmonella* expansion needs to be considered with an intervention strategy that increases neutrophils in circulation. It may be important to establish an optimal neutrophilia for the greatest *Salmonella* reduction with minimal neutrophil-stimulated tissue damage that, in itself, could encourage *S*. Typhimurium virulence factor-induced inflammation (36).

As regulatory and public scrutiny necessitates the judicious use of antibiotics in food animals (39, 40), the need for antibiotic alternatives in animal production intensifies. Naturally occurring biotherapeutics engineered for pharmaceutical application offer an alternative to antibiotic usage, especially for prophylactic or possibly metaphylactic administration during periods of anticipated stress and host susceptibility. Through the general activation of innate immune defenses, immunostimulants may provide effective pathogen reduction or elimination with broad application against bacteria and viruses that pose a food safety threat or that negatively impact animal health. Our results suggest that prophylactic use of Ad5-G-CSF in swine could decrease subclinical or clinical disease by microorganisms that are targeted by neutrophils.

## Author Contributions

SB, BB, CL, HA, DM, and MK conceived and designed experiments. SB, BB, CL, HA, IL, and DM performed the experiments. SB, BB, CL, HA, and MK wrote and edited the manuscript.

## Conflict of Interest Statement

Mention of trade names or commercial products in this article is solely for the purpose of providing specific information and does not imply recommendations or endorsement by the U.S. Department of Agriculture. The USDA is an equal opportunity provider and employer. The authors declare that the research was conducted in the absence of any commercial or financial relationships that could be construed as a potential conflict of interest.
